# Editorial: Global excellence in intensive care medicine and anesthesiology: North America

**DOI:** 10.3389/fmed.2022.1039307

**Published:** 2022-09-23

**Authors:** Mariam ElSaban, Rahul Kashyap

**Affiliations:** ^1^Mohammed Bin Rashid University of Medicine and Health Sciences, Dubai, United Arab Emirates; ^2^Department of Anesthesiology and Perioperative Medicine, Mayo Clinic, Rochester, MN, United States; ^3^Medical Director Research, WellSpan Health, York, PA, United States; ^4^Global Clinical Scholars Research Trainee, Harvard Medical School, Boston, MA, United States

**Keywords:** anesthesiology, North America, COVID-19, perioperative, EHR, telehealth, intensive care medicine

We enthusiastically present the special edition research series in Frontiers in Medicine, “Global Excellence in Intensive care medicine and anesthesiology: North America”. This series aims to highlight the global collaborations in intensive care medicine that form the cornerstone of scientific development. The history of intensive care medicine has been rife with turbulent times, the most recent being the COVID-19 pandemic. Hospitals and health systems around the globe adapted to the increasing demand for healthcare need. This has undoubtedly placed an unimaginable strain on healthcare workers but has also fostered an environment for innovation. Out-of-the-box thinking and innovation in critical care medicine are the mainstay themes in this series.

Song et al. present an analysis of the first multicenter registry of CytoSorb use (a blood purification method) in COVID-19 patients on ECMO. The FDA gave CytoSorb emergency use authorization (EUA) for the indication to hemoadsorb inflammatory cytokines in patients with COVID-19 in respiratory failure (1, 2). The ICU mortality was 30.8% by 153 days among 52 studied patients. The rates of ICU discharge exceeded mortality after starting therapy. Song et al. conclude that CytoSorb therapy shows a potential therapeutic benefit in specific subsets of COVID-19 patients.

Complementing the latest therapies to treat severe COVID-19, intensive care units worldwide also necessitated tools for improved communication (3). The use of telehealth expanded during this time. Laudanski et al. studied the perceptions and communications of remote teams in Tele-critical care medicine (tele-CCM) across 16 intensive care units. These teams consisted of a multi-professional group of medical doctors (eMD), registered nurses (eRN), and respiratory therapists (eRT). Through their comprehensive study, Laudanski et al. inspected close to 40,000 engagements that occurred using tele-CCM services, analyzed communication patterns, and compared variations between members of the healthcare teams. Their analyses will help guide policy making when planning for the resources required to maintain the Tele-CCM service.

Critical care and anesthesiology practices are not limited to the operating room or intensive care unit only. The pre-operative and post-operative settings, as well as the post-critical care illness journeys, are just as essential. The following two articles delve into these domains.

Undergoing surgery is undoubtedly a stressful time for patients. The sympathetic nervous system's adrenergic response is augmented. Pain and stress are some of the most common disstressors. Often, pharmacological therapies are utilized in the peri-operative period to blunt these responses. While they are useful, non-pharmacologic interventions are often overlooked. Ginsberg et al. review the use of perioperative music medicine (PMM) to reduce pain and stress around surgery. They also look into the effects of PMM on heart rate variability (HRV) as a marker of surgery-induced sympathetic stress response. Perioperative music medicine is a promising addition to surgical practices, with low barriers to adoption and a wide range of patient benefits.

The hospital discharge papers do not mean the end of a patient's journey with a critical illness. Ahmed et al. set out to explore patient-centered outcomes in electronic medical records following critical illness in the elderly population. This pilot study showed that “free text” in the EHR contains essential data that give insight into patients' lives after recovering from critical illness. They show that the majority of the studied charts (86%) had enough information to paint a picture of the patient's health state pre and post-ICU admission. These promising findings will pave the path to conducting larger-scale studies to define important patient-centered outcomes and establish ways to extract them from EMR (4, 5). The goal is for these outcomes to be used in critical care research to complement traditional outcomes like mortality rates. Data mining presents a functional tool to use in this endeavor (6).

In summary, the research series “Global excellence in intensive care medicine and anesthesiology: North America” reflects on relevant contemporary questions and seeks innovative solutions to improve patient outcomes. Future volumes of the series will continue to highlight topics of interest in critical care and anesthesiology communities.

## Author contributions

ME conceived the idea and drafted the manuscript. RK conceived the idea, critically reviewed the paper, and interpretation of the work. Both authors contributed to the article and approved the final submitted version.

## Conflict of interest

The authors declare that the research was conducted in the absence of any commercial or financial relationships that could be construed as a potential conflict of interest.

## Publisher's note

All claims expressed in this article are solely those of the authors and do not necessarily represent those of their affiliated organizations, or those of the publisher, the editors and the reviewers. Any product that may be evaluated in this article, or claim that may be made by its manufacturer, is not guaranteed or endorsed by the publisher.
